# Integrative Treatment Strategies for Chronic Back Pain: A Literature Review with Clinical Recommendations

**DOI:** 10.3390/ijerph22020289

**Published:** 2025-02-15

**Authors:** Nina H. Russin, Alexis M. Koskan, Lesley Manson

**Affiliations:** College of Health Solutions, Arizona State University, 425 N. 5th St., Phoenix, AZ 85004, USA; alexis.koskan@asu.edu (A.M.K.); lesley.manson@asu.edu (L.M.)

**Keywords:** chronic back pain, biopsychosocial model, brief behavioral interventions, lifestyle modification, psychoeducation, older adults, primary care

## Abstract

Problem: Chronic back pain (CBP) is a major cause of disability, contributing significantly to healthcare costs and primary care visits. Pharmacotherapy alone is insufficient in managing CBP. Integrated behavioral health interventions that include psychoeducation are critical for a more holistic, sustainable treatment of CBP. Objectives: This review explores CBP treatments that includes psychoeducation as part of patient care. Methods: In the Fall of 2024, the first author searched Google Scholar, PubMed, and Scopus using search terms related to chronic back pain and integrated behavioral interventions to increase patients’ self-efficacy to manage CBP. The team included articles in the review that were published more recently and seminal articles in the field of managing CBP. Results: Given the complex biopsychosocial factors influencing CBP, and the individualized nature of each patient’s pain experience, patient psychoeducation should include a multimodal approach, which may include cognitive behavioral strategies to address pain, pain neuroscience education, and education related to lifestyle behaviors such as physical activity, sleep, nutrition, and stress management. Patient education and behavioral interventions integrated within primary care can significantly improve patient engagement and self-reported improvements in pain intensity, functionality, and quality of life. Conclusion: Psychoeducation is foundational for integrative programs aimed at managing CBP.

## 1. Introduction

### 1.1. Chronic Back Pain and Its Burden

Chronic back pain (CBP) lasting for more than 3 months is a leading cause of disability in the United States, affecting 80% of adults over a lifetime [1,2]. It is the second most common reason for primary care visits [3] and the fourth leading contributor to chronic healthcare costs [4]. According to the 2021 Global Burdens of Diseases, in 2020, low back pain affected 619 million individuals, and it is projected to increase to 843 million by 2050 [5]. Additionally, low-back-pain-related disability increased globally by 54% from 1990 to 2015 [6]. CBP is especially concerning among older adults, with a prevalence in the United States estimated at 32% [7] and increasing annually [8]. Additionally, among older adults with new-onset back pain, only 25% resolve the issue within a year, with 75% progressing to chronic pain [9]. Within this cohort, CBP has been associated with comorbid arthritis, stress, anxiety, and depression, and these psychosocial factors significantly affect pain intensity and quality of life [10].

Although widely used to treat and manage CBP, pharmacotherapeutic treatments, including oral medications and nerve blocks, are increasingly scrutinized due to their cost and limited efficacy [11]. A retrospective analysis of over 100 million insurance claims estimated individual costs between USD 6,590 and 10,156, contributing to an economic burden of USD 624.8 billion in the U.S. [12]. Additional concerns surrounding pharmacotherapy for older adults include drug–drug interactions when opioids are prescribed for pain relief [13,14], age-related changes in drug metabolism, and compliance issues related to cognitive decline [15]. Given these figures, there is a growing need to reassess treatment strategies that heavily depend on pharmacotherapy [16].

### 1.2. Biopsychosocial Model of Chronic Pain

The biopsychosocial model of chronic pain proposes that pain lasting for more than three months arises from an interaction between physiological, psychological, and environmental factors, all of which must be addressed for effective management [17]. Unlike acute pain, which is typically physical in nature and serves a protective purpose [18], chronic pain is influenced by stress, anxiety, depression, environmental factors, and support networks [19,20]). The biopsychosocial model evolved from early theories, including Selye’s stress adaptation theory [21] and Wall and Melzack’s Gate Control and Neuromatrix theories [22]. Chronic pain, distinct from acute pain, should be viewed from the perspective of illness rather than injury or disease [17]. Effective management therefore requires addressing both intrinsic (e.g., emotional state) and extrinsic (e.g., lifestyle) contributors, with strategies including psychoeducation and brief behavioral interventions [23,24,25,26]. Further, integrative treatments that include psychoeducation, such as cognitive behavioral therapy (CBT), acupuncture, and chiropractic care, have proven to be more effective and sustainable for treating chronic pain than pharmacotherapy alone [27,28]. Therefore, the purpose of this review is to explore CBP treatments that include psychoeducation as part of patient care.

## 2. Materials and Methods

To explore CBP management and treatments that include psychoeducation, the first author conducted multiple searches of PubMed, Scopus, and Google Scholar in the Fall of 2024. The initial search of Google Scholar and PubMed utilized the following framework for search terms and their categorization:

1. Problem: chronic back pain in older adults within primary care
2. Interventions: psychoeducation, brief behavioral interventions; lifestyle modification
3. Clinical recommendations: screening and patient self-reporting of pain

The following search strategies were employed: Search 1: “chronic back pain”; Search 2: “non-pharmacologic treatments” AND “chronic back pain”; Search 3: “psychoeducation” AND “chronic back pain”; Search 4: “patient self-management” AND “chronic back pain”; Search 5: “patient provider interactions” AND “chronic back pain”; Search 6: “physical activity” AND “chronic pain” AND “sleep hygiene” AND “chronic pain” AND “stress, anxiety, depression” AND “chronic back pain”; Search 7: “screening for anxiety” AND “depression” AND “chronic back pain”; and Search 8: “patient self-evaluation” AND “chronic back pain”. Although the search focused on recent research, the first author also included what she considered to be foundational studies.

The second search utilizing Google Scholar, PubMed, and Scopus included the following search terms: “biopsychosocial model” AND “chronic back pain”, “drug interactions” AND “chronic back pain”, “chronic back pain” AND “older adults”, “chronic back pain” AND “self-management”, and “brief behavioral interventions” AND “chronic back pain”. The authors examined behavioral interventions in the context of chronic pain self-management, including psychoeducation, cognitive behavioral therapy, motivational interviewing, attention shifting, relaxation techniques, and their delivery methods (e.g., in-person, digital, print, hybrid) as well as the context (e.g., functional rehabilitation, pain symptom management, etc.). Finally, the review and clinical recommendations include lifestyle modifications such as physical activity, sleep improvements, and socialization within the framework of psychoeducation. No time frame restrictions were applied to ensure the comprehensive coverage of seminal research on psychosocial interventions in chronic pain management.

## 3. Results

Given the complex biopsychosocial factors influencing CBP [29], and the individualized nature of each patient’s pain experience [30], patient CBP treatment programs should adopt a multimodal approach that includes psychoeducation [31,32,33,34]. Psychoeducation in terms of cognitive behavioral therapy (and offshoots such as acceptance and commitment therapy, and mindfulness-based stress reduction), motivational interviewing, and other similar evidence-based psychoeducation interventions can serve as an umbrella for framing and engaging patients. In addition, the treatment plan should include pain self-management techniques such as gentle movement and resilience-building exercises [35,36]. As part of pain management, physiotherapy is valuable for improving flexibility, range of motion, and for correcting muscular imbalances that can both lead to and sustain chronic back pain [37]. These strategies may be integrated into primary care practices that involve behavioral health consultants (BHCs), who may work in separate practices or alongside primary care providers in fully integrated practices.

### 3.1. Psychoeducation for Patient Buy-In

Psychoeducation serves a variety of purposes, from helping patients to understand the link between chronic pain and affective disorders to the co-creation of strategies for lifestyle improvements related to chronic pain, such as sleep hygiene, physical activity, and nutrition [38,39]. Psychoeducation can help to engage patients in evidence-based behavioral interventions by explaining the rationale behind these processes [40,41]. Teach back, an education strategy in which a patient repeats back to the provider information about a treatment strategy in their own words [42], ensures that the patient comprehends the treatment strategy adequately.

### 3.2. Motivational Interviewing to Engage Patients in Pain Self-Management

Motivational interviewing based on reflective listening can be a good way to engage patients and encourage their own evaluation of their specific pain management needs. The MI process is based on the OARS acronym, representing the four key interviewing skills which include (1) asking open-ended questions, (2) affirming, (3) reflective listening, and (4) summarizing [43]. MI interventions are typically brief and do not require any special licensing; however, before implementation, individuals must undergo MI training and practice. There are numerous resources for MI trainers, including the Motivational Interviewing Network of Trainers [44] and academic organizations. While MI might not work well as a stand-alone treatment [45,46], it is useful in combination with other therapeutic modalities, such as pain neuroscience education, physical activity, cognitive behavioral, and relaxation strategies [47].

### 3.3. Cognitive Behavioral Strategies for Chronic Pain Management

Cognitive behavioral strategies assist patients in recognizing and addressing maladaptive thought patterns, such as catastrophizing and all-or-nothing thinking, that influence their pain experience [48,49,50]. In addition to the original CBT methodology developed by Beck in the 1970s, a new wave of CBT includes mindfulness-based stress reduction (MBSR), which integrates meditation practices with cognitive approaches, and acceptance and commitment therapy (ACT), which encourages individuals to focus on value-based actions [51]. These techniques, delivered individually or in groups, help patients to build self-efficacy and improve adherence to treatment plans [52]. CBT is one of the most widely used approaches to address chronic pain in multimodal programs that include brief behavioral interventions and lifestyle modification.

Homework plays an important role in some types of cognitive behavioral therapy, as it serves to reinforce ideas learned in the clinic by asking the patient to associate maladaptive thought processes with their own behavior [53]. For example, a patient might be asked to choose one or two types of maladaptive thought patterns from a list and describe how they might utilize different, more productive thought strategies (i.e., not catastrophizing, not predicting future pain events, etc.). The key here is engaging patients during in-clinic sessions to help them understand the benefit of such practices, and how challenging maladaptive thought patterns can help reduce their subjective experience of chronic pain. This might entail explaining the bidirectional relationship between anxiety, depression, and chronic pain.

### 3.4. Pain Neuroscience Education

Pain neuroscience education is a layman’s approach to the physiology and psychology of chronic pain, to help patients understand the bidirectional relationship behind emotional states and chronic pain, along with why physiological processes driving chronic pain differ from those causing acute pain [30,54,55]. Pain neuroscience education helps patients understand the process of pain perception, emphasizing that pain is influenced by both psychological and physiological factors [30,55,56]. Key to this understanding is the concept of central sensitization, where the central nervous system becomes hypersensitive over time [55]. Internal and external stressors, such as anxiety, depression, and poor sleep, can exacerbate pain perception. Educating patients about these mechanisms enables them to develop personalized self-management strategies, improving both physical and emotional well-being [19]. Neuroscience pain education may be a worthwhile addition to other interventions, such as physical activity [57], since together they have been shown to produce moderate short-term improvements in reducing disability [58].

### 3.5. Physical Activity

Chronic back pain is influenced by both physiological and psychological factors, such as fear avoidance of physical activity [30,59,60,61]. The fear-avoidance model, applied to CBP, explains how fear of movement can reduce physical activity and increase disability [62]. Encouraging engagement in rehabilitative exercises paired with education on pain pathophysiology can address these fears and, therefore improve recovery [33,63]. Physical activity, crucial for restoring daily functioning, is widely agreed upon, though recommendations vary [64,65].

Prescribing physical activity for older adults may be carried out by physicians, physiatrists, physical therapists, or personal trainers with specialized training and experience working with this population. Particularly for older adults, it is important to focus on function for physical independence and quality of life [64]. According to the American College of Sports Medicine (ACSM), the following are four considerations when designing an exercise program: (1) All individuals lose proprioception (the ability of the body to sense its position in space) when they age. Therefore, simple balance exercises such as standing with their feet together, a tandem stance, and a single leg stance should be part of the physical activity program. (2) Postural muscle groups should be stressed with exercises such as heel and toe stands. (3) Be wary of movements that involve excessive forward bending, which can lead to compression fractures in persons who have osteoporosis or osteopenia. (4) Include functional activities such as sit-to-stand that will help individuals perform their activities of daily living (ADLs) more easily [65].

The ACSM recommends ‘prescribing’ exercise using the F.I.T.T format, which segments each activity according to frequency, intensity, time, and type [65]. A simple prescription form, available on the Exercise is Medicine website, contains this information and a place for the provider’s signature, which may further illustrate the importance of physical activity as medicine [66].

### 3.6. Sleep Hygiene

Disordered sleep among older adults has been associated with greater levels of depression and physical pain [67], suggesting that the presence of disordered sleep may exacerbate CBP and sleep disorders. Given this bidirectional relationship, non-pharmacologic strategies to improve sleep quality can significantly impact the quality of life of people living with CBP [68,69]. Cognitive Behavioral Therapy for Insomnia (CBT-I), a non-pharmacological treatment, includes strategies such as sleep restriction, stimulus control, relaxation, and cognitive restructuring to improve sleep hygiene and manage disruptive thought patterns [70]. These interventions target the psychological factors influencing sleep, enhancing long-term sleep quality and potentially reducing chronic pain. In some cases, even simple suggestions may be helpful, such as limiting daytime naps, tai chi exercise, art therapy, soothing music, relaxation, and meditation exercises [71].

### 3.7. Nutrition

While the link between CBP and diet may not be immediately apparent, educating patients on proper nutrition is crucial for maintaining a healthy metabolic profile. Multiple studies support a significant relationship between obesity and chronic pain [72,73], with research suggesting that up to a third of patients with low back pain are overweight or obese [74]. In addition to forcing the spine to accommodate a greater load, their weight distribution and center of gravity may change, making the ability to walk more difficult [75]. The Western diet’s energy-dense foods also contribute to low back pain due to increased inflammatory factors such as IL-6 and CRP [76]. Readily available patient education resources are available through the Academy of Nutrition and Dietetics [77] and the USDA [78] to enhance nutrition support and education for patients experiencing chronic pain.

While treatment for morbid obesity is beyond the scope of this report, there are simple strategies that can help to engage patients in planning to form healthier eating habits. The Stages of Change, also known as the Transtheoretical Model of Behavior Change [79], describes a continuum of readiness for behavior change, from pre-contemplation (not willing to consider a behavior change) through active change and maintenance, and adds a temporal dimension to the change process, although progress is not necessarily linear. To aid patients in making achievable, realistic, and sustainable nutrition-related lifestyle changes, providers can help patients develop a series of SMART goals (goals that are Specific, Measurable, Attainable, Relevant, and Time-bound) along the continuum of change. Additionally, helping patients to understand that lifestyle changes may occur slowly and over time can help to make changing dietary behaviors more manageable. For patients with more extensive needs, providers might consider referral to a registered dietician.

### 3.8. Relaxation Techniques for Pain Management

Relaxation techniques are an important component of CBP self-management [80,81]. In primary care, effective behavioral interventions include deep breathing, progressive muscle relaxation, and guided imagery, which help regulate sympathetic arousal [51,82]. In a busy clinic, deep breathing may be the simplest technique to guide patients who are unfamiliar with relaxation practices. It requires minimal instruction, and can be carried out when the patient is either supine or in a comfortable seated position. These techniques aim to reduce pain perception by calming the body’s response to stress, ultimately enhancing patients’ ability to manage chronic pain. For those who are willing to invest additional time in learning relaxation practices outside of the clinic, mindfulness meditation, which helps practitioners to maintain present focus and detached observation [83], may be helpful.

### 3.9. Socialization

Patients living with chronic health conditions, including low back pain, are more likely to experience social isolation [84], which is predictive of poor treatment outcomes [85]. Social isolation can stem from both physical limitations, such as impaired mobility, and emotional challenges like anxiety and depression [84]. Social isolation has been associated with a significantly increased risk of premature mortality among older adults [86]. Addressing this bidirectional relationship by fostering social connections, in-person or via social media, may improve the quality of life and functionality for these patients [85]. Warm lines, which may be run by peers or trained non-professionals, provide a means for individuals experiencing social isolation to contact for support outside of traditional office hours [87]. Warm lines differ from hotlines in that they are not focused on severe emotional issues (e.g., suicidality), but rather simply provide a human connection to those who feel lonesome or are experiencing social isolation [87].

### 3.10. Integrating Behavioral Health for Chronic Back Pain Within Primary Care

Treating individuals with CBP will require implementing a number of the above-mentioned strategies, based on a patient’s needs, to address the physical and psychological influences of this debilitating condition. It can be challenging for primary care physicians and specialists, already under time-constrained pressures and administrative duties, to incorporate these interventions into their patient care. Therefore, it may be preferable to divert behavioral health for pain management to a separate provider, particularly behavioral health consultants, who work at the practice and are integrated into the patient’s care team [88,89]. Bringing in an expert in behavioral health has similar benefits to physical therapy in addressing the physical symptoms of chronic pain: it expands the spectrum of care. While specific strategies for psychoeducation and brief behavioral interventions do not require special licensing, BHCs have the advantage of experience with addressing affective comorbidities such as stress, anxiety, and depression, and they may be more skilled in suggesting targeted behavioral health treatment strategies, depending on the patient’s needs. In addition, BHCs can take the lead in creating patient education materials which may take the form of print-based, digital, or video-based education materials (e.g., exercise videos to manage acute pain flare-ups), to assist patients in adhering to treatment recommendations outside of the clinic.

While fully integrating behavioral health consulting within a practice represents a significant investment, it has distinct advantages over other options [90]. In the U.S., BHCs who are licensed clinical social workers or licensed psychologists are reimbursable through Medicare and most major insurance companies, with separate codes for different types of interventions [90,91]. In addition, patients are more likely to be compliant with visits to behavioral health providers if those visits take place within the primary care practice concurrent with the visit to the primary care provider (PCP) [92]. The PCP can introduce the patient to the BHC via a ‘warm handoff’ and can also consult with the BHC informally through hallway conversations [92], which improves continuity of care. For older adults who no longer drive and may need to arrange transportation to the clinic, having both their physical and behavioral needs met in a single location during one office visit greatly simplifies this process.

### 3.11. Clinical Recommendations for Psychosocial Assessment and Patient Satisfaction

Clinical guidelines recommend psychological assessment for co-occurring stress, anxiety, and depression in patients with chronic pain to predict treatment outcomes [93]. Simple tools, such as the Generalized Anxiety Disorder 7 (GAD-7) questionnaire and Patient Health Questionnaire 9 (PHQ-9), help screen for anxiety and depression [54]. In cases involving traumatic injury, screening for PTSD using the four-question Primary Care PTSD screen (PC-PTSD), is recommended [94]. Both adverse childhood experiences (ACEs) and PTSD have been associated with the transition from acute to chronic pain [95], with PTSD accompanying chronic pain in up to 54% of cases [96], and ACEs impacting pain severity via emotional dysregulation [97]. The ACE-10 screening tool is limited to adverse events within the family and/or household (and does not consider other social determinants) [98], but it is validated and short enough to administer in a primary care setting. Additionally, assessing pain intensity, functionality, and quality of life is vital before and during treatment, using brief tools like the pain, enjoyment of life, and general activity (PEG) questionnaire [99].

An empathetic provider–patient relationship fosters trust, enabling effective shared decision-making and the co-creation of personalized treatment plans [80,100]. Brief behavioral interventions, such as cognitive behavioral therapy (CBT), motivational interviewing, and utilizing the Transtheoretical model [101], help patients to identify barriers to change and improve treatment engagement. CBT helps patients to recognize cognitive distortions affecting pain perception [52], while motivational interviewing encourages reflection on lifestyle changes [43], enhancing patient adherence to therapeutic recommendations.

It is also important to keep track of changes in patients’ subjective ratings of pain, their ability to perform ADLs, and changes in their self-rated quality of life. The Pain, Enjoyment of life, and General activities (PEG) questionnaire is an efficient, validated tool for primary care settings [99,102,103]. Additionally, the Brief Pain Inventory (BPI) provides a comprehensive assessment, with proven internal validity [104], especially for non-malignant chronic back pain.

While healthcare providers acknowledge the importance of patient education in enhancing self-efficacy [105], significant variability exists in how educational materials are delivered [106]. Physician-prescribed materials, written for general audiences, may include pain management strategies for acute flare-ups [107]. Web-based interventions, particularly those using cognitive behavioral therapy to reduce catastrophizing, show promise [108]. Additionally, exercise videos and lifestyle modification resources may reinforce office-based physical therapy. Digital and print-based educational materials have been shown to improve motivation and patient empowerment [108]. Providing patients with a ‘toolbox’ of strategies gives patients a sense of control over their chronic pain.

## 4. Conclusions

Chronic back pain remains a significant cause of disability, and while integrative approaches are widely endorsed, gaps in systematic self-management strategies with a focus on patient psychoeducation persist. Psychoeducation is crucial in helping patients understand the multifaceted nature and drivers of pain, empowering them to engage in effective self-management. It is critical to pair psychoeducation with other interventions (e.g., encouragement to engage in physical activity, nutrition counseling, and taught relaxation techniques) to address CBP more effectively. Healthcare providers are key in promoting these strategies, enhancing patient motivation and self-efficacy in managing their pain. Clinicians, including BHCs, can offer psychoeducation while prescribing lifestyle modifications to most effectively empower patients to manage their chronic back pain and thus improve their quality of life.

## Data Availability

There are no data to report, only publicly available peer-reviewed articles.
